# Establishment of liquid biopsy procedure for the analysis of circulating cell free DNA, exosomes, RNA and proteins in colorectal cancer and adenoma patients

**DOI:** 10.1038/s41598-024-78497-x

**Published:** 2024-11-06

**Authors:** Andrea Čeri, Anita Somborac-Bačura, Marija Fabijanec, Andrea Hulina-Tomašković, Marko Matusina, Dijana Detel, Donatella Verbanac, Karmela Barišić

**Affiliations:** 1 Department of Medical Biochemistry and Haematology, University of Zagreb Faculty of Pharmacy and Biochemistry, Zagreb, 10000 Croatia; 2 Centre for Applied Medical Biochemistry, University of Zagreb Faculty of Pharmacy and Biochemistry, Zagreb, 10000 Croatia; 3 Department of Medical Chemistry, Biochemistry and Clinical Chemistry, University of Rijeka Faculty of Medicine, Rijeka, 51000 Croatia

**Keywords:** Circulating tumour DNA, Colorectal cancer, Exosomes, Liquid biopsy, Methodology challenges, microRNA, Medical research, Molecular medicine, Gastrointestinal cancer

## Abstract

Liquid biopsy has an underexplored diagnostic potential in colorectal cancer (CRC). Sufficient quantity and quality of its elements (circulating cell-free DNA (ccfDNA), exosomes and exosomal RNA) are essential for accurate results. The present study aims to establish the optimal protocol for handling liquid biopsy samples. Samples were obtained by collecting peripheral blood from colorectal adenoma patients in CellSave tubes. Plasma was separated within six hours using differential centrifugation and aliquots stored at – 20/– 80 °C until further processing. Three methods for isolation of ccfDNA, and two combinations of kits for isolation of exosomes and exosomal RNA were tested. The quality and quantity of ccfDNA isolates were evaluated. Exosomes were characterised by determining size, concentration, and total and specific protein content. Expression of chosen microRNAs, miR-19a-3p and miR-92-3p, which have been implicated in CRC progression, were determined. The vacuum-column-based kit showed the highest quantities of isolated ccfDNA (P-value < 0.001). Kits for exosome isolation significantly differed in size (P-value = 0.016), concentration (P-value = 0.016) and protein content (P-value = 0.016). There was no significant difference in expressions of miR-19a-3p (P-value = 0.219) and miR-92a-3p (P-value = 0.094) between the two isolation kits. The new, adapted protocol described, enables simultaneous analysis of multiple elements when investigating potential biomarkers of CRC.

**Supplementary Information:**

The online version contains supplementary material available at 10.1038/s41598-024-78497-x.

## Introduction

Colorectal cancer (CRC) results from the accumulation of multiple cellular modifications, both genetic and epigenetic, that manifest in the transformation of colonic epithelial cells into invasive and aggressive adenocarcinomas^1,2^. Due to disease complexity and to improve patient outcomes, research of new optimal biomarkers for early detection, progression, personalised treatment selection, and monitoring response to therapy is necessary^3,4^.

Liquid biopsy is a non-invasive and repeatable method for studying biomarkers in the body fluids of patients with cancer, which eliminates the need for invasive tissue biopsies, offering a cost and time reduction, but also making it a safer and more convenient option for patients, particularly in cases where traditional tissue biopsies are not feasible or where the tumour evolves rapidly^4,5^. It allows the detection and analysis of tumour-derived components, such as circulating tumour DNA (ctDNA), circulating tumour cells (CTCs), and exosomes, which reflect tumour characteristics, genetic alterations, and functional changes^6^. Therefore, liquid biopsy makes a promising tool in the molecular profiling of tumours, and real-time monitoring of disease progression, providing valuable information for personalized cancer treatment based on the patient’s unique tumour characteristics, and predicting treatment response, leading to more effective and targeted cancer care^7,8^. Factors that can affect the samples before analysis, mainly due to contamination of the sample with cellular content after cell lysis, are the choice of sample collection tube and preservative, the time required from sampling to processing, storage and transport conditions of the sample, processing conditions (speed, duration and temperature of centrifugation steps during sample processing), repeated freeze-thaw cycles and, finally, methods for extracting elements of liquid biopsy, as well as storage of final isolates^9,10^.

Circulating cell-free DNA (ccfDNA) are fragments of DNA released into the bloodstream by the apoptosis of hematopoietic cells in healthy individuals, but also from tumour cells, increasing the overall level of ccfDNA at the expense of ctDNA, especially in patients with advanced disease and distant metastases^11,12^. Apoptosis digests DNA into mono- (cca. 166 base pairs (bp)), di- (cca. 332 bp), and tri- (cca. 498 bp) nucleosomal units, whereas ctDNA is shorter, often reaching as low as 90 bp^13^. ctDNA may possess critical genetic alterations that can provide particular markers for detecting CRC tumorigenesis^14^. Personalised treatment options can be adjusted by panel next-generation sequencing (NGS)-based evaluation of driver mutations in ctDNA, but the newest research shows the further potential to improve disease monitoring, increase the sensitivity of minimal residual disease identification, and detection of cancers at earlier stages by its multi-modal assessment trough evaluation of ctDNA fragment-omics, nucleosome modifications and methylation patterns^15^.

Exosomes, these small (30–150 nm) bilayer and stable extracellular vesicles, are not just a by-product of malign processes, but key players in cancer development and metastasis^8,16,17^. Exosomes derived from CRC contain metastatic factors, signalling molecules, lipid rafts, and their associated elements. For example, specific intracellular CRC proteins were found in the exosomes of CRC patients, a discovery that could revolutionize the early detection of CRC^18–20^. Moreover, in exosomes of CRC patients, levels of proteins involved in the remodelling of the extracellular matrix, intercellular communication, cell signalling, increased vascular permeability, and tumour-promoting inflammation are increased, while levels of proteins involved in immune evasion, complement binding, cell adhesion, and tumour growth are decreased^21^. Finally, exosomes may contain different RNA molecules, including mRNA, microRNA, long non-coding RNA, and circular RNA. Specific exosomal microRNA were found to be elevated in invasive metastatic tumours and associated with poor prognosis, a finding that could potentially guide treatment decisions^8,22^.

However, the current knowledge on the potential and significance of ccfDNA and exosome analysis in managing patients with CRC needs to be revised for their full application in clinical settings due to limited sensitivity and specificity, which requires additional, comprehensive research^4,8^. Despite the available literature data on the pre-analytical variables that affect the quality of liquid biopsy samples, including sample handling and the application of different extraction methods for individual elements, the lack of standardization presents a challenge for clinical implementation, especially since sufficient quantity and quality of each component is of crucial importance for obtaining accurate results in further analysis^9,10,13,23,24^. Therefore, this study, with its comprehensive design and meticulous examination of samples obtained from colorectal adenoma patients, aims to establish an optimal protocol for handling liquid biopsy samples, to acquire suitable isolates (ccfDNA, exosomes and microRNA) for further assessment of specific biomarkers for colorectal adenoma and CRC.

## Results

### ccfDNA isolation and analysis

**Fig. 1:**
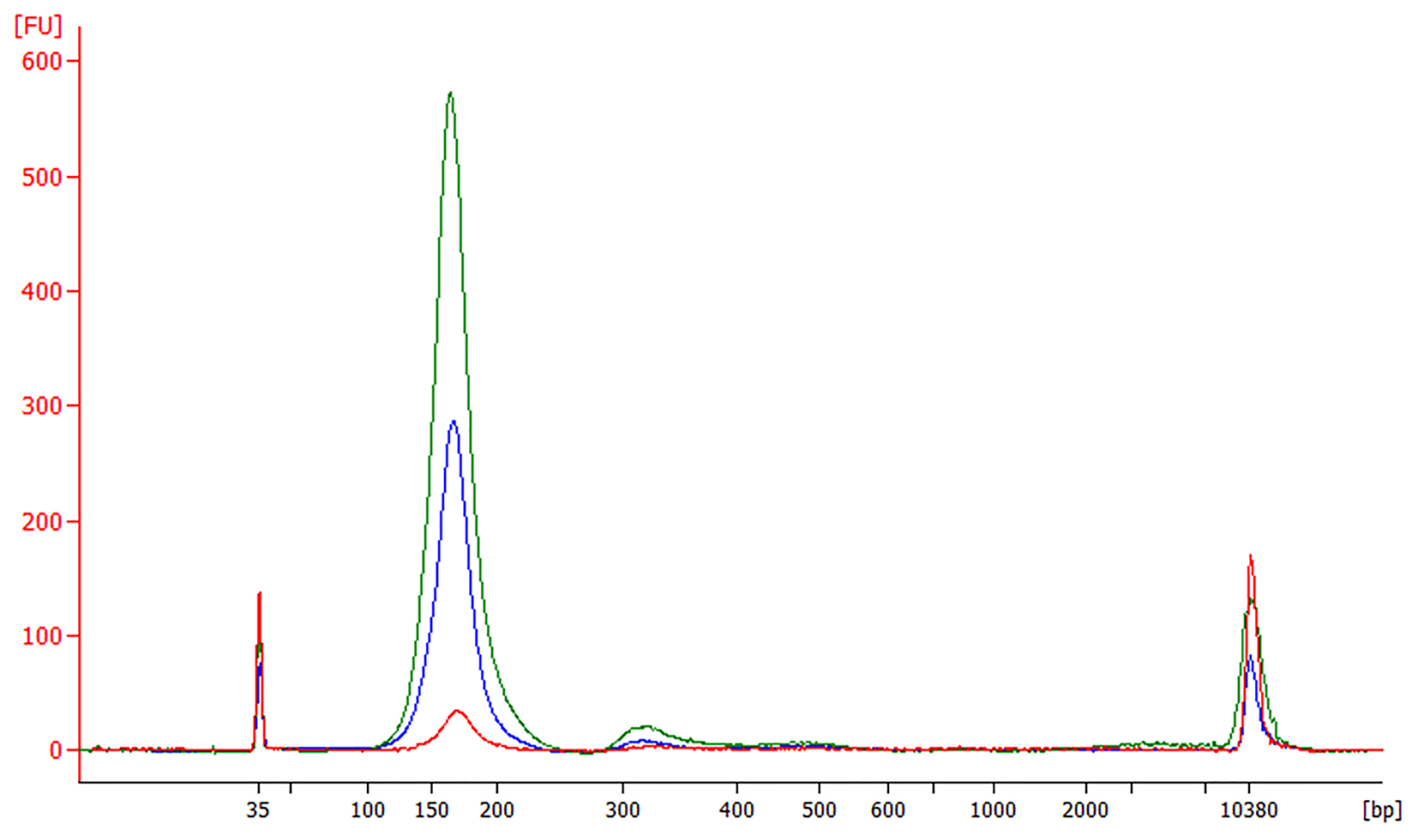
Examples of electropherograms of ccfDNA isolates obtained on the Bioanalyzer 2100 using High Sensitivity DNA Kit. Isolates of sample number 5 with the highest quantity of ccfDNA per mL of plasma obtained using all three methods are shown. Electropherogram corresponding to sample isolated by NucleoSpin cfDNA XS Kit (Macherey-Nagel, Düren, Germany) is shown in red, QIAamp^®^ ccfDNA/RNA Kit (Qiagen, Hilden, Germany) is shown in blue, and QIAamp^®^ Circulating Nucleic Acid Kit (Qiagen, Hilden, Germany) is shown in green. In addition to the peaks corresponding to the lower (35 bp) and upper (10,380 pb) markers, peaks corresponding to the mononucleosome (~ 165 bp) and dinucleosome (~ 320 bp) are also visible.

**Fig. 2:**
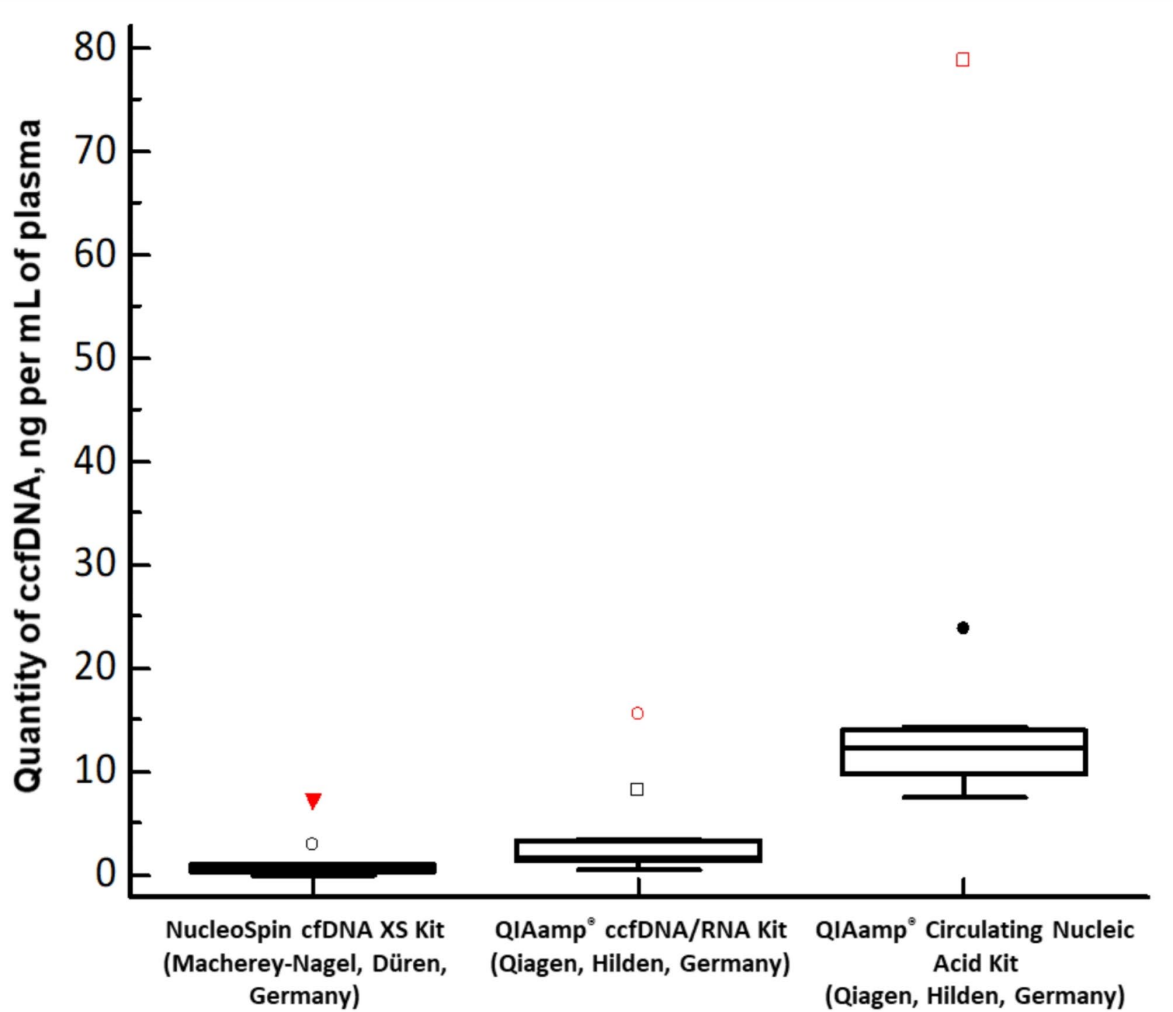
Quantities of isolated ccfDNA depending on the isolation method used (*n* = 11). There were significant differences in the quantities of ccfDNA in isolates between the three kits (P-value < 0.001). P-value was obtained using Friedman test for paired samples in MedCalc Statistical Software, v22.020 (MedCalc Software Ltd, Ostend, Belgium). Red colour indicates the isolates of sample number 5 with the highest quantity of isolated ccfDNA using each isolation method.

ccfDNA was successfully isolated from all 11 plasma samples and isolates were analysed by automated electrophoresis and fluorimetric measurement to determine the quality and quantity of ccfDNA. The fragment corresponding to ccfDNA was found by automated electrophoresis in all 11 QIAamp^®^ Circulating Nucleic Acid Kit (Qiagen, Hilden, Germany) isolates (additional dinucleosomal peak was visible in four isolates), nine QIAamp^®^ ccfDNA/RNA Kit (Qiagen, Hilden, Germany) isolates (additional dinucleosomal peak was visible in two isolates; HMW DNA contamination was suspected in two isolates), and two NucleoSpin^®^ cfDNA XS Kit (Macherey-Nagel, Düren, Germany) isolates (Table 1. and Fig. 1.). The quantities of isolated ccfDNA per mL of plasma were significantly different among the three methods (P-value < 0.001), showing the highest amounts using the QIAamp^®^ Circulating Nucleic Acid Kit as compared to the Qiagen spin-column-based method and the NucleoSpin^®^ cfDNA XS Kit, the later one resulting in the lowest quantities (Fig. 2.).

**Table 1:** Obtained electrophoretic peaks corresponding to ccfDNA depending on the isolation method used.

Sample number	Peak size corresponding to ccfDNA, bp		
	NucleoSpin cfDNA XS Kit (Macherey-Nagel, Düren, Germany)	QIAamp^®^ ccfDNA/RNA Kit (Qiagen, Hilden, Germany)	QIAamp^®^ Circulating Nucleic Acid Kit (Qiagen, Hilden, Germany)
1	/	/	174
2	/	179	172
3	/	/	178
4	/	172	173
5	165	167 and 317	164 and 322
6	/	168	170
7	/	175	183
8	/	170	181 and 374
9	/	190	162
10	/	146	174 and 329
11	163	166 and 329	163 and 316

ccfDNA, circulating cell-free DNA; bp, base pairs.

### Exosome isolation and characterisation

**Fig. 3:**
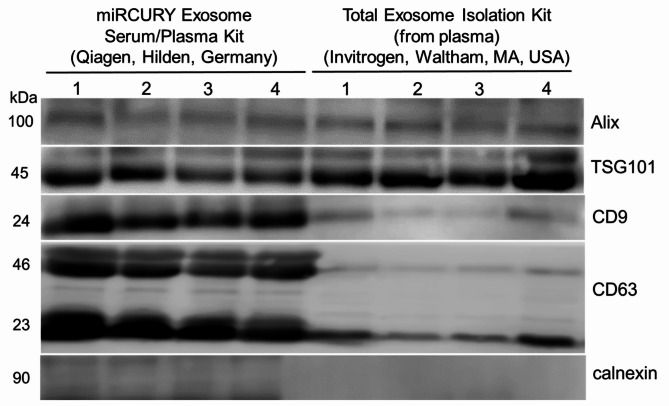
Characterization of exosomes derived from plasma samples of patients with colorectal adenoma using the Western blotting analysis. Representative blots of four samples of exosomes isolated by miRCURY Exosome Serum/Plasma Kit (Qiagen, Hilden, Germany) and paired four samples isolated by Total Exosome Isolation Kit (from plasma) (Invitrogen, Waltham, MA, USA) are shown. Blots were cropped to highlight the area of interest; the original images of the blots and positive outcome for calnexin are provided in Supplementary Figs. S1 and S2.

The results of the size, concentration, and protein content of exosomes isolated using two different kits from seven samples are presented in Table 2. The obtained size, concentration, and protein content of exosomes significantly differed between isolation kits. Larger exosomes were obtained with the Total Exosome Isolation Kit (from plasma) (Invitrogen, Waltham, MA, USA) than the miRCURY Exosome Serum/Plasma Kit (Qiagen, Hilden, Germany) (P-value = 0.016). However, the miRCURY Exosome Serum/Plasma Kit (Qiagen, Hilden, Germany) yielded a significantly higher concentration of exosomes (P-value = 0.016) and total exosomal proteins (P-value = 0.016) than the Total Exosome Isolation Kit (from plasma) (Invitrogen, Waltham, MA, USA). Further characterization of exosomes from four paired samples by Western blotting analysis is presented in Fig. 3. In the samples of exosomes isolated with the miRCURY Exosome Serum/Plasma Kit (Qiagen, Hilden, Germany), the presence of exosomal markers CD9 and CD63 was demonstrated, as well as the presence of cytosolic proteins TSG101 and Alix. In contrast, the characteristic band for calnexin was absent, proving the absence of endoplasmic reticulum. In the samples of exosomes isolated with the Total Exosome Isolation Kit (from plasma) (Invitrogen, Waltham, MA, USA), the exosomal markers CD9 and CD63 were present in meagre amounts.

**Table 2:** The size, concentration and protein content of exosomes isolated with two different kits (*n* = 7). Data are presented as median (interquartile range). P-values were obtained using the Wilcoxon test for paired samples in MedCalc Statistical Software, v22.020 (MedCalc Software Ltd, Ostend, Belgium).

	miRCURY Exosome Serum/Plasma Kit (Qiagen, Hilden, Germany)	Total Exosome Isolation Kit (from plasma) (Invitrogen, Waltham, MA, USA)	*P*-value
mean size (nm)	29.9 (26.6 – 42.5)	86.4 (77.0 – 97.4)	0.016
concentration (particles/mL) ×10^13^	379.1 (123.0 – 426.5)	4.7 (0.1 – 11.3)	0.016
protein content (ng/mL)	21.1 (16.2 – 22.1)	0.6 (0.2 – 1.0)	0.016

### microRNA isolation and analysis

**Fig. 4:**
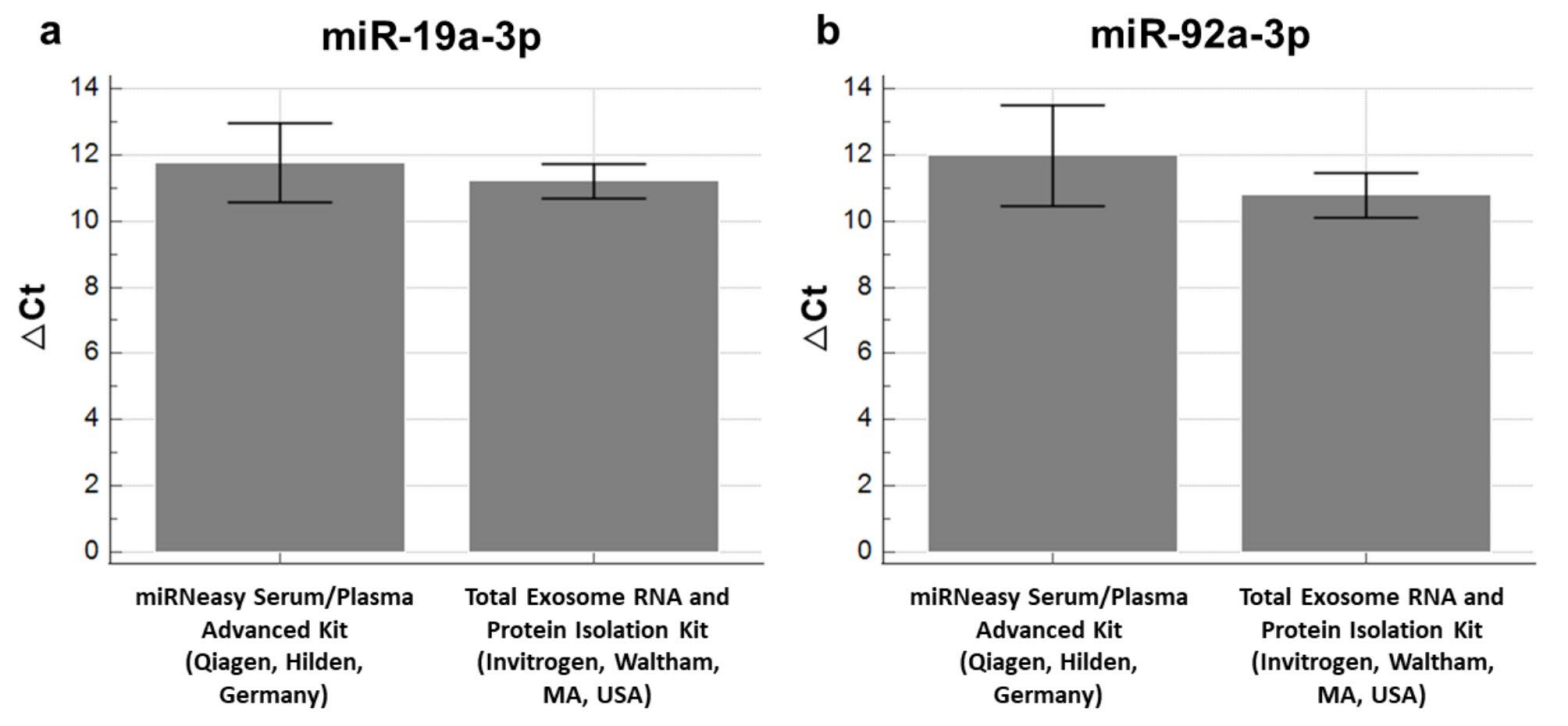
Expressions of miR-19a-3p (a) and miR-92a-3p (b) are shown as ΔCt in samples isolated from exosomes by two different methods (*n* = 6). There were no significant differences in the expressions of miR-19a-3p (P-value = 0.219) and miR-92a-3p (P-value = 0.094) between the two methods. P-values were obtained using the Wilcoxon test for paired samples in MedCalc Statistical Software, v22.020 (MedCalc Software Ltd, Ostend, Belgium).

To compare the performance of miRNeasy Serum/Plasma Advanced Kit (Qiagen, Hilden, Germany) and Total Exosome RNA and Protein Isolation Kit (Invitrogen, Waltham, MA, USA), microRNA expression analysis was carried out. Seven paired samples were analysed, and the chosen microRNAs were detected in six of them. There was no significant difference in ΔCt for miR-19a-3p (P-value = 0.219) and miR-92a-3p (P-value = 0.094) between the two methods (Fig. 4.).

## Discussion

The work being described presents a detailed and unique protocol for the proper assessment of liquid biopsy samples. The aim was to overcome many pre-analytical challenges and obtain samples that are suitable for further ccfDNA, exosomal RNA and protein analysis from both colorectal adenoma and CRC patients. The used CellSave Preservative tubes contain Na_2_EDTA and a cell preservative that stabilize cells in the sample for up to 96 h, which allows simultaneous analysis of CTCs from the same sample. Although there are other tubes marketed for the liquid biopsy sampling that use different preservation chemistries that affect the final plasma dilutions^9^, such as Cell-Free DNA BCT tube (Streck, La Vista, NE, USA), PAXgene Blood ccfDNA tube (BD, Franklin Lakes, NJ, USA) or cf-DNA/cf-RNA Preservative tube (Norgen, Thorold, Ontario, Canada), CellSave Preservative tube is both IVD registered and compatible with CellSearch^®^ technology, the gold standard for counting circulating tumour cells, which facilitates implementation in clinical practice.

To the best of our knowledge, there are no published studies that describe sample processing for the simultaneous analysis of different elements of liquid biopsy, which is the main strength of this research. Sample collection, processing, storage, and thawing were all taken into account, and several methods for the isolation of liquid biopsy elements were tested to find the optimal one. However, due to the restricted initial blood sample volume, differences in blood collection tubes and pre-treatment procedures (different storage conditions, processing time, and temperature) were not evaluated, which is a limitation of this study. Only one tube option and the optimal pre-treatment procedure that corresponded to all tested DNA/exosome/RNA isolation kits were tested.

Regarding ccfDNA isolation, there are > 40 commercially available kits for manual and automated isolation that show variability in recovery efficiency, size, and reproducibility and can be divided according to basic principles to the ones that use binding of DNA to silica gel membranes, magnetic particles, or organic chemicals^13^. In this study, three kits were tested where the vacuum-column-based QIAamp^®^ Circulating Nucleic Acid Kit (Qiagen, Hilden, Germany) proved to be the most suitable as it provided the highest quantities of ccfDNA and visible electrophoretic peaks corresponding to ccfDNA without suspected HMW DNA contamination, compared to other tested methods. Ungerer et al., analysed 20 previously published studies comparing commonly used ccfDNA isolation methods and concluded an excellent recovery efficiency of the same kit. However, previously published studies did not use the same ccfDNA quantification method, and only a few included verifying ccfDNA isolates, making their further comparison difficult^13^. Maass et al., conducted a comprehensive comparison of multiple ccfDNA quantification methods and automated electrophoresis on two platforms. While their study did not use the same preservation tubes and isolation methods as the presented study, they concluded that the Qubit^®^ dsDNA HS Assay Kit demonstrated superior performance and reproducibility in estimating ccfDNA quantity. Similarly, automated chip electrophoresis on Bioanalyzer 2100 using High Sensitivity DNA Kit was found to provide optimal separation of ccfDNA and HMW DNA^9^. In this study, the same high-sensitivity DNA quantification method and automated chip electrophoresis with the same high-sensitivity reagents suitable for the analysis of short DNA fragments were adopted. This meticulous approach was also applied in the study by Polatoglou et al.^25^, where two of the three isolation kits included in our study were compared and showed similar performance. Based on these rigorous findings and previously published data, the vacuum-column-based QIAamp^®^ Circulating Nucleic Acid Kit (Qiagen, Hilden, Germany) was selected for further processing of liquid biopsy samples from CRC patients. This kit consistently produced the desired quality and quantity for downstream applications such as digital PCR and NGS.

The International Society for Extracellular Vesicles recently released an updated version of available approaches for studying extracellular vesicles from different sources, including body fluids^26^. According to these guidelines, pre-analytical variables and the method of separation must be described in detail and characterization should include the quantitative measures of the source of extracellular vesicles, quantification of particles, protein and/or lipid content, but also a demonstration of the presence of the characteristic components and absence of the non-vesicular, co-isolated components. There is no gold standard for the isolation of exosomes, although different methods based on the characteristic size, density, charge, and surface composition of extracellular vesicles can be used, namely differential ultracentrifugation, density gradients, size-exclusion chromatography, ultrafiltration, polymer-induced precipitation, immunoaffinity, microfluidics-based techniques, asymmetric flow field-flow fractionation, etc. Each method shows certain advantages and disadvantages, providing differences in recovery and specificity^23,26,27^. Lately, the most widely used diagnostic methods are based on precipitation and microfluidics due to their broad applicability and high efficiency^27^. Moreover, the composition of the cargo and the extent of contamination with plasma proteins can be influenced by the isolation method of exosomes from plasma^23^. In the context of clinical application, the isolation method’s primary requirements are high yield and purity. In this study, exosomes were isolated from freshly separated and frozen plasma using two different kits. The size and particle concentration were determined immediately after isolation, and the isolates were characterized by the determination of two transmembrane proteins (CD9 and CD63), two cytosolic proteins (TSG101 and Alix), and one protein associated with other intracellular compartments (calnexin). The results of the exosome characterization revealed the meticulousness of the research, with the miRCURY Exosome Serum/Plasma Kit (Qiagen, Hilden, Germany) yielding a higher quantity of exosomes as compared to the Total Exosome Isolation Kit (from plasma) (Invitrogen, Waltham, MA, USA). Despite the larger size of exosomes obtained with the Invitrogen method, the concentration and protein content of exosomes were notably higher with the miRCURY Exosome Serum/Plasma Kit (Qiagen, Hilden, Germany), instilling the reliability of our findings. In addition, the Western blotting results proved the high purity of exosomes obtained with the miRCURY Exosome Serum/Plasma Kit (Qiagen, Hilden, Germany), with the presence of the characteristic markers, the absence of endoplasmic reticulum marker, and the suitability of isolates for further analyses of exosomal content (e.g., proteins and RNA molecules).

No significant difference was observed between the exosomal RNA isolation methods in the obtained ΔCt for miR-19a-3p and miR-92a-3p. While the use of a specific RNA isolation kit depended on the exosome isolation method, both methods allowed further isolation of RNA from as little as 200 µL of exosome isolates while being both simple and rapid. Wright et al.^28^ found that the miRNeasy Serum/Plasma Advanced Kit (Qiagen, Hilden, Germany), compared to three other methods, allowed the isolation of total RNA enriched with RNA smaller than 200 nucleotides, was more user-friendly and showed less variation between fresh and frozen samples. Tang et al., demonstrated better performance of the combination of Total Exosome Isolation Kit (from plasma) (Invitrogen, Waltham, MA, USA) and Total Exosome RNA and Protein Isolation Kit (Invitrogen, Waltham, MA, USA) for protein and RNA extraction as compared to kits from other manufacturers, but comparison to the combination of miRCURY Exosome Serum/Plasma Kit (Qiagen, Hilden, Germany) and miRNeasy Serum/Plasma Advanced Kit (Qiagen, Hilden, Germany) for protein and RNA extraction was not described. They also concluded that the initial exosome isolation method could potentially affect the result of RNA and/or protein isolation^29^. In this study, a specific combination of exosome and RNA extraction methods were used due to the small amount of sample available, being one of the limitations of the study. Based on the better performance of miRCURY Exosome Serum/Plasma Kit (Qiagen, Hilden, Germany) for exosome isolation, miRNeasy Serum/Plasma Advanced Kit (Qiagen, Hilden, Germany) was selected for further RNA isolations.

The practical application of liquid biopsy in CRC assessment is promising, but also challenging due to the low analyte content, low sensitivity, specificity and reliability of individual analytes, the rapidly increasing number of commercial products available for sample processing, and the lack of systematic external quality control of laboratory procedures^8^. However, optimisation and standardisation of sample processing and analysis procedures are prerequisites for obtaining comparable and reproducible results of all further analyses. Integration of all pre-analytical and analytical methods, including simultaneous investigation of potential biomarkers of disease in different targets (CTCs, ctDNA and exosomal RNA and proteins) into clinical validation protocol for clinical trials is crucial for the future application of liquid biopsy as part of routine practice in the treatment of the CRC.

## Materials and methods

### Liquid biopsy sample collection and plasma preparation

Patients with colorectal adenoma were recruited during regular clinical examinations at the University Hospital Centre “Sestre milosrdnice”. Liquid biopsy samples were obtained by collecting peripheral blood in two 10 mL CellSave Preservative tubes (Menarini Silicon Biosystems, Bologna, Italy), stored and transported at 4 °C to the laboratory. The samples were processed in a UV pre-sterilised laboratory, with sterile DNase/RNase-free tubes, and filter pipette tips. Plasma was separated within six hours of sample collection by differential centrifugation in a pre-cooled refrigerated centrifuge LISA (AFI, Château-Gontier, France) using a swing-out rotor to ensure gentle centrifugation, except for centrifugation at 16,000 × *g* which was performed using a fixed-angle rotor. After first centrifugation at 1900 × *g* and 4 °C for 10 min, 3.3 mL of plasma was separated into a new tube and centrifuged again at 3000 × *g* and 4 °C for 15 min to obtain a cell-free supernatant with low platelet content, which was stored in aliquots until the isolation of exosomes and further extraction of exosomal RNA. All remaining supernatant after first centrifugation was transferred into another tube and centrifuged at 16,000 × *g* and 4 °C for 10 min, after which new supernatant was stored in aliquots until the extraction of ccfDNA. The sample was rejected for further processing in case of visible haemolysis after the first centrifugation step. All plasma aliquots were stored at – 20 °C for short-term storage (up to a month) or – 80 °C for long-term storage. Further processing was performed within six months of plasma separation.

The written informed consent was obtained from all patients. The study was conducted in accordance with the tenets of the Declaration of Helsinki and approved by the Ethics Committee for Experimentation of the University of Zagreb Faculty of Pharmacy and Biochemistry (approval no. 251-62-03-19-29) and Ethics Committee of the University Hospital Centre “Sestre milosrdnice”, Zagreb, Croatia (approval no. EP-19243/17-6).

### Isolation of ccfDNA

Three kits for the isolation of ccfDNA from previously stored plasma aliquots of 11 colorectal adenoma patients were tested and compared based on the quality and quantity of obtained isolates. These included two spin-column-based isolation kits and one vacuum-column-based kit. The isolation of ccfDNA using the NucleoSpin^®^ cfDNA XS Kit (Macherey-Nagel, Düren, Germany) was performed from a thawed 700 µL aliquot of plasma according to the manufacturer’s guidelines using the enclosed proteinase K and a final elution volume of 20 µL. The isolation of ccfDNA using the QIAamp^®^ ccfDNA/RNA Kit (Qiagen, Hilden, Germany) was performed from a thawed aliquot (2.0 – 3.5 mL) of plasma according to the manufacturer’s guidelines with the repeated centrifugation step after protein precipitation and a final elution volume of 20 µL. Centrifugations of enclosed Midi columns were performed using a refrigerated centrifuge LISA (AFI, Château-Gontier, France) with a swing-out rotor to ensure gentle centrifugation and even passage of the sample through the column. The isolation of ccfDNA using the QIAamp^®^ Circulating Nucleic Acid Kit (Qiagen, Hilden, Germany) was performed using the QIAvac vacuum system, consisting of QIAvac 24 Plus, QIAvac Connecting System and a vacuum pump (Qiagen, Hilden, Germany), from another aliquot (2.0 – 3.5 mL) of plasma according to the manufacturer’s guidelines and after centrifugal separation of the cryoprecipitate formed by thawing the aliquot, with a final elution volume of 50 µL. Centrifugation of provided NucleoSpin^®^ cfDNA XS columns, RNeasy MinElute^®^ Spin columns and QIAamp^®^ Mini columns was performed on a MiniSpin^®^ centrifuge (Eppendorf, Hamburg, Germany). All isolates were stored at – 20 °C until further analysis.

To verify the presence of fragments corresponding to ccfDNA and the absence of high-molecular weight DNA (HMW DNA) contamination (> 1000 bp), all isolates were analysed by automated chip electrophoresis on Bioanalyzer 2100 using High Sensitivity DNA Kit (Agilent Technologies, Santa Clara, CA, USA). Concentrations of ccfDNA were determined by fluorometric measurement on DS-11 FX (DeNovix, Wilmington, DE, USA) using Qubit^®^ dsDNA HS Assay Kit (Invitrogen, Waltham, MA, USA). The quantities of isolated ccfDNA per mL of plasma were calculated.

### Isolation of exosomes

For the isolation of exosomes, two commercially available kits were compared, namely miRCURY Exosome Serum/Plasma Kit (Qiagen, Hilden, Germany) and Total Exosome Isolation Kit (from plasma) (Invitrogen, Waltham, MA, USA). Both were based on the precipitation of exosomes from plasma by capturing water molecules, which forces less-soluble components (i.e. exosomes) out of solution, allowing their collection by centrifugation. After thawing on ice, plasma aliquots from seven colorectal adenoma patients were centrifuged at 3000 × *g* and + 4 °C for 10 min to remove debris and cryoprecipitate. An initial volume of 600 µL of plasma was used for all isolations. When using the miRCURY Exosome Serum/Plasma Kit (Qiagen, Hilden, Germany), the first centrifugation step at 3000 × *g* and room temperature was performed for 10 min, while the remaining steps were performed according to the manufacturer’s guidelines. The final volume of the isolates was 270 µL. Before isolation with the Total Exosome Isolation Kit (from plasma) (Invitrogen, Waltham, MA, USA), an additional centrifugation step at 10,000 × *g* at room temperature for 20 min was performed to remove debris from the plasma. Incubation with enclosed proteinase K was included, while the remaining steps were performed according to the manufacturer’s guidelines. The isolate was obtained by resuspension of the final pellet with 270 µL of resuspension buffer provided with the Total Exosome RNA and Protein Isolation Kit (Invitrogen, Waltham, MA, USA).

To demonstrate the yield and purity of exosomes obtained by each kit, the size and quantity of particles, protein content, the presence of the characteristic exosomal components, and the absence of the co-isolated components, were determined. 50 µL of freshly obtained isolates diluted 20× in 1× sterile phosphate buffer saline (Merck, Darmstadt, Germany) was used for determination of the size and concentration of exosomes by the dynamic light scattering method on the Zetasizer Nano ZS (Malvern Panalytical, Malvern, UK). Aliquots stored at – 80 °C from four paired samples were used to prove the presence and purity of the isolated exosomes by Western blotting analysis. The protein concentration in all samples was determined by spectrophotometric method with bicinchoninic acid and CuSO_4_ (Sigma-Aldrich, St. Louis, MO, USA). Exosomes were lysed with radioimmunoprecipitation buffer. Samples were prepared using 2× Laemmli buffer with β-mercaptoethanol and proteins were separated by 8% or 10% sodium dodecyl sulfate (SDS)-polyacrylamide gel electrophoresis using the Mini-PROTEAN^®^ Electrophoresis System (Bio-Rad Laboratories, Hercules, CA, USA). Trans-Blot^®^ SD System (Bio-Rad Laboratories, Hercules, CA, USA) was used to transfer the proteins to the Immobilon^®^ polyvinylidene fluoride membrane (Merck-Millipore, Burlington, MA, USA). After blocking with 5% non-fat dry milk (Bio-Rad Laboratories, Hercules, CA, USA) in Tris-buffered saline (Sigma-Aldrich, Steinheim, Germany) containing 0.1% Tween-20 (TBST) (Amersham Biosciences, Amersham, UK) at room temperature for 1 h, membranes were incubated at 4 °C overnight with primary antibodies. Antibodies used to detect specific target proteins are shown in Table 3. The blots were washed with TBST at room temperature. Corresponding bands were detected by chemiluminescence using SignalFire Elite ECL Reagent (Cell Signaling, Danvers, MA, USA) and ImageQuant^®^ LAS 4000 mini (GE Healthcare, Chicago, IL, USA).

**Table 3:** Antibodies used in Western blotting analysis for the detection of specific target proteins for quality assessment of isolated exosomes.

Catalogue number	Manufacturer details	Target protein	Purpose of use	Dilution
10626D	Invitrogen, Waltham, MA, USA	CD9	Exosomal marker	1:500
sc-5275	Santa Cruz Biotechnology, Dallas, TX, USA	CD63	Exosomal marker	1:200
sc-7964	Santa Cruz Biotechnology, Dallas, USA	TSG101	Cytosolic protein	1:200
MA1-83977	Invitrogen, Waltham, MA, USA	Alix	Cytosolic protein	1:500
MA3-027	Invitrogen, Waltham, MA, USA	Calnexin	Endoplasmic reticulum marker (impurity marker)	1:500
ab97023	Abcam, Cambridge, UK	Primary antibodies	Detection	1:10,000

### Isolation of exosomal microRNA and expression analysis

Exosomal RNA was isolated from seven paired exosome isolates on the same day as the exosome isolations, according to manufacturer’s guidelines. Isolation using miRNeasy Serum/Plasma Advanced Kit (Qiagen, Hilden, Germany) was performed from 200 µL of exosomes isolated by miRCURY Exosome Serum/Plasma Kit (Qiagen, Hilden, Germany) with the final elution volume of 20 µL. Isolation using the Total Exosome RNA and Protein Isolation Kit (Invitrogen, Waltham, MA, USA) was performed from 200 µL of exosomes isolated using an Total Exosome Isolation Kit (from plasma) (Invitrogen, Waltham, MA, USA) with the final elution volume of 100 µL. All isolates were stored at – 80 °C until further analysis. The cDNA for microRNA expression analysis was obtained using the miRCURY RT LNA Kit (Qiagen, Hiden, Germany). Expressions of miR-19a-3p and miR-92a-3p were assessed with the miRCURY LNA SYBR GREEN PCR System (Qiagen, Hilden, Germany) on the 7500 Real-Time PCR System (Applied Biosystems, Foster City, CA, USA), with the UniSp6 serving as the internal control.

### Statistical analysis

Statistical analysis was performed using MedCalc Statistical Software, v22.020 (MedCalc Software Ltd, Ostend, Belgium). To evaluate three ccfDNA isolation kits, the amounts of ccfDNA in paired isolates (*n* = 11) were analysed using the Friedman test for paired samples that allows comparison of three data sets. The exosome and RNA isolation kits were evaluated by analysing sizes, concentrations, and protein contents in paired exosome isolates (*n* = 7), and △Ct values obtained from expression assays in paired RNA isolates (*n* = 6), respectively, using the Wilcoxon test for paired samples. These statistical tests were chosen due to the small number of samples included in the analyses. A P-value < 0.050 was considered statistically significant.

## Electronic supplementary material

Below is the link to the electronic supplementary material.

Supplementary Material 1

## Data Availability

The raw data generated during the current study are available from the corresponding author on reasonable request.

## Abbreviations

bp: Base pairs
ccfDNA: Circulating cell-free deoxyribonucleic acid
ctDNA: Circulating tumour deoxyribonucleic acid
CRC: Colorectal cancer
CTC: Circulating tumour cell
HMW DNA: High-molecular weight deoxyribonucleic acid
NGS: Next-generation sequencing
TBST: Tris-buffered saline containing 0.1% Tween-20
