# Author Correction: Establishment of liquid biopsy procedure for the analysis of circulating cell free DNA, exosomes, RNA and proteins in colorectal cancer and adenoma patients

**DOI:** 10.1038/s41598-024-80214-7

**Published:** 2024-11-19

**Authors:** Andrea Čeri, Anita Somborac-Bačura, Marija Fabijanec, Andrea Hulina-Tomašković, Marko Matusina, Dijana Detel, Donatella Verbanac, Karmela Barišić

**Affiliations:** 1 Department of Medical Biochemistry and Haematology, University of Zagreb Faculty of Pharmacy and Biochemistry, Zagreb, 10000 Croatia; 2 Centre for Applied Medical Biochemistry, University of Zagreb Faculty of Pharmacy and Biochemistry, Zagreb, 10000 Croatia; 3 Department of Medical Chemistry, Biochemistry and Clinical Chemistry, University of Rijeka Faculty of Medicine, Rijeka, 51000 Croatia

Correction to: *Scientific Reports* 10.1038/s41598-024-78497-x, published online 06 November 2024

The Funding section in the original version of this Article was omitted.

The Funding section now reads: “This research was funded by the Croatian Science Foundation, grant number IP‐2019‐04‐4624 (project “Genetic, protein and RNA profiling of colorectal cancer using liquid biopsy”) and supported by the project FarmInova (KK.01.1.1.02.0021) funded by the European Regional Development Fund.”

The original Article has been corrected.

