# Supplementary material for: Establishment of liquid biopsy procedure for the analysis of circulating cell free DNA, exosomes, RNA and proteins in colorectal cancer and adenoma patients
Source: Sci Rep. 2024 Nov 6;14:26925. doi: 10.1038/s41598-024-78497-x (PMC11541997; doi:10.1038/s41598-024-78497-x)
Supplement: Supplementary file 1 — Supplementary Material 1 [file 41598_2024_78497_MOESM1_ESM.docx]

**Establishment of liquid biopsy procedure for the analysis of circulating cell free DNA, exosomes, RNA and proteins in colorectal cancer and adenoma patients**

Andrea Čeri^*1^, Anita Somborac-Bačura^1^, Marija Fabijanec^2^, Andrea Hulina Tomašković^1^, Marko Matusina^1^, Dijana Detel^3^, Donatella Verbanac^1^, Karmela Barišić^1^

^1^Department of Medical Biochemistry and Haematology, University of Zagreb Faculty of Pharmacy and Biochemistry, Zagreb, 10000, Croatia

^2^Centre for Applied Medical Biochemistry, University of Zagreb Faculty of Pharmacy and Biochemistry, Zagreb, 10000, Croatia

^3^Department of Medical Chemistry, Biochemistry and Clinical Chemistry, University of Rijeka Faculty of Medicine, Rijeka, 51000, Croatia

*[andrea.ceri@pharma.unizg.hr](mailto:andrea.ceri@pharma.unizg.hr)

**
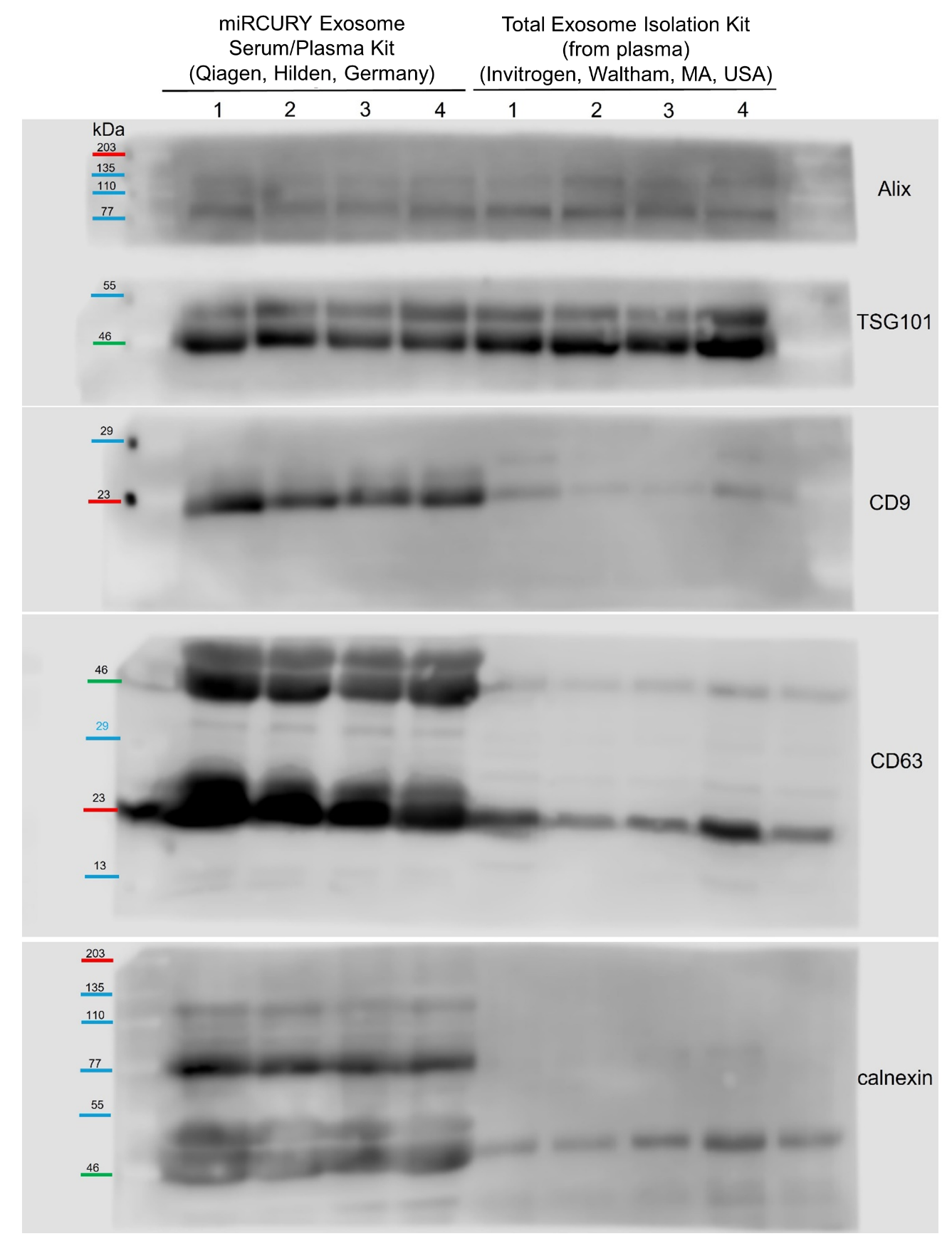
**

**Supplementary Figure S1.** Characterization of exosomes derived from plasma samples of patients with colorectal adenoma using the Western blotting analysis. Expressions of transmembrane (CD9, CD63) and cytosolic proteins (Alix, TSG101) as well as calnexin as a negative marker of exosomes were analysed. Representative blots of four samples of exosomes isolated by miRCURY Exosome Serum/Plasma Kit (Qiagen, Hilden, Germany) and paired four samples isolated by Total Exosome Isolation Kit (from plasma) (Invitrogen, Waltham, MA, USA) are shown.

**
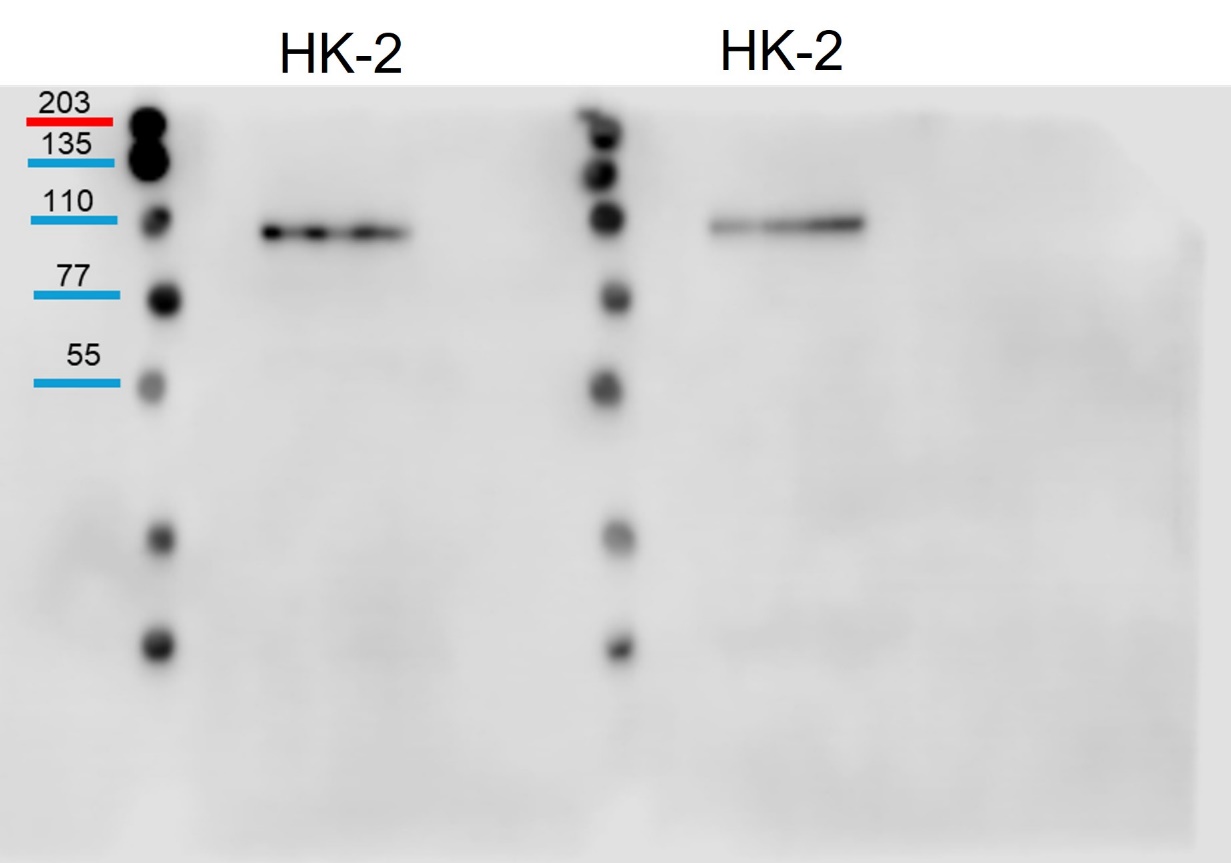
**

**Supplementary Figure S2.** Representative blots of two dilutions of calnexin antibody tested on Human renal proximal tubular cells (HK-2), obtained from the American Type Culture Collection (ATCC^®^, CRL-2190™, Rockville, MD, USA).
